# Great debate: myocardial infarction after cardiac surgery must be redefined

**DOI:** 10.1093/eurheartj/ehae416

**Published:** 2024-09-16

**Authors:** Mario Gaudino, Allan S Jaffe, Milan Milojevic, Yader Sandoval, Philip J Devereaux, Kristian Thygesen, Patrick O Myers, Jolanda Kluin

**Affiliations:** Department of Cardiothoracic Surgery, Weill Cornell Medicine, New York, NY, USA; Departments of Cardiology and Laboratory Medicine and Pathology and Wayne and Kathryn Presiel Professor of Cardiovascular Disease Research, Mayo Clinic, Rochester, MN, USA; Department of Cardiac Surgery and Cardiovascular Research, Dedinje Cardiovascular Institute, Belgrade, Serbia; Department of Cardiothoracic Surgery, Erasmus MC, Dr. Molewaterplein 40, 3015 GD Rotterdam, The Netherlands; Minneapolis Heart Institute, Abbott Northwestern Hospital, Minneapolis Heart Institute Foundation, Minneapolis, MN, USA; Department of Medicine, McMaster University, Hamilton, ON, Canada; Department of Health Research Methods, Evidence, and Impact, McMaster University, Hamilton, ON, Canada; Population Health Research Institute, Perioperative & Surgery, Hamilton, ON, Canada; Department of Cardiological Medicine, Aarhus University Hospital, Aarhus, Denmark; Department of Cardiac Surgery, CHUV-Center Hospitalier Universitaire Vaudois, Lausanne, Switzerland; Department of Cardiothoracic Surgery, Erasmus MC, Dr. Molewaterplein 40, 3015 GD Rotterdam, The Netherlands

**Keywords:** Perioperative myocardial infarction, Definition, Cardiac surgery, Myocardial injury

## Abstract

**Graphical Abstract:**
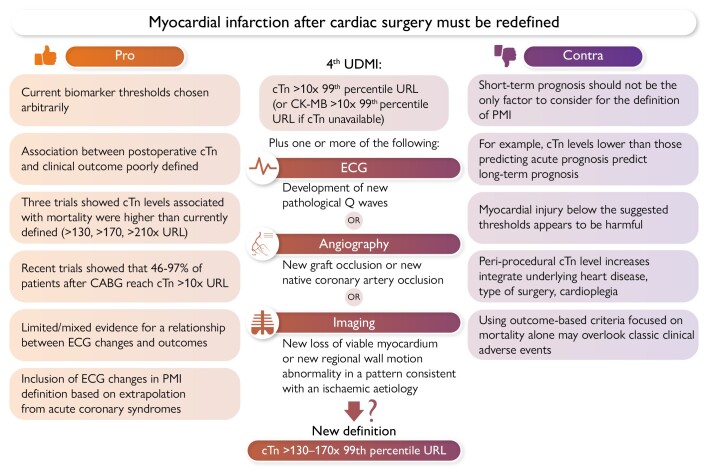
Pros and cons of redefining peri-operative myocardial infarction (PMI) to cardiac troponin (cTn) levels of at least 130–170× 99th percentile upper reference limit (URL). CABG, coronary artery bypass grafting; CK–MB, creatine kinase–myocardial band; ECG, electrocardiogram; MI, myocardial infarction; UDMI, universal definition of myocardial infarction.

Pros and cons of redefining peri-operative myocardial infarction (PMI) to cardiac troponin (cTn) levels of at least 130–170× 99th percentile upper reference limit (URL). CABG, coronary artery bypass grafting; CK–MB, creatine kinase–myocardial band; ECG, electrocardiogram; MI, myocardial infarction; UDMI, universal definition of myocardial infarction.

## Introduction

https://orcid.org/0000-0002-5942-4727KluinJolanda
Department of Cardiothoracic Surgery, Erasmus Medical
Center, Dr. Molewaterplein 40, 3015 GD
Rotterdam, The Netherlands

It is important to detect peri-operative myocardial infarction (PMI) after cardiac surgery. First and most importantly to enable identifying patients that are post-operatively at risk of ongoing infarction and might need either a percutaneous coronary intervention (PCI) or a reoperation. These are most often patients after coronary bypass surgery. But patients after other cardiac procedures can also experience ischaemia due to coronary artery problems (e.g. calcific emboli following decalcification in aortic stenosis/aortic valve replacement procedures, or injury of the circumflex artery due to a stitch deep in the annulus of the posterior mitral valve leaflet, placed to implant a mitral ring in mitral valve repair surgery). A second reason to detect PMI following cardiac surgery is for quality control. In most national registries, PMI after cardiac surgery is an item of the quality control registry. Third, in studies, especially in those that compare a surgical procedure [e.g. coronary artery bypass grafting (CABG) or surgical aortic valve replacement] with a transcatheter procedure (e.g. PCI or transcatheter aortic valve replacement), it is important to distinguish patients with a PMI from those that have ‘general myocardial injury’ due to the surgical procedure itself. Examples of myocardial injury due to the surgery itself include mechanical manipulation and cannulation, cardiopulmonary bypass, cardioplegic arrest, ischaemia–reperfusion injury, peri-operative tachyarrhythmias, incisions/stitches in the myocardium, ablation (maze procedure), and myocardial resection (e.g. myectomy in hypertrophic cardiomyopathy surgery).^1^ All these procedure-related mechanisms can induce cardiac enzyme/biomarker release. In most studies comparing CABG with PCI, PMI is part of the combined endpoint of major adverse cardiac and cerebrovascular events. Examples like the Evaluation of XIENCE versus Coronary Artery Bypass Surgery for Effectiveness of Left Main Revascularization (EXCEL) trial show the importance of a correct definition of PMI.^2,3^ In this trial, the investigators used an enzyme-based definition of PMI [creatine kinase–myocardial band (CK–MB) above 10× upper reference limit (URL)], and it was implied that this detrimentally impacted on those with CABG procedures. Lastly, PMI might impact long-term outcome. One might say that a definition of PMI is only/most valid when a PMI, defined accordingly, is associated with long-term mortality after cardiac surgery. However, mortality is the worst, though not the only important outcome. An association with morbidity or low quality of life is important as well. Furthermore, this reasoning will lead to a high threshold to reach the definition of a PMI since only the larger PMIs are associated with mortality.^4^ This will result in underdiagnosis of the smaller though possibly treatable PMIs that can benefit from post-operative PCI or reoperation.

Thus, a too low cut-off value for cardiac troponin (cTn) elevation will result in a high number of false-positive PMIs that can put patients in danger of undergoing a not-indicated post-operative coronary angiography or reoperation and can falsely derogate CABG or other cardiac surgery procedure outcomes in trials comparing surgery with transcatheter procedures. On the other hand, a too high cut-off value for cTn elevation will result in a high number of false negatives, resulting in erroneously not offering patients a post-operative coronary angiography or reoperation and a failure to identify institutes with lower quality of care.

Currently, several definitions of PMI exist.

1. The 4th Universal Definition (4UD) of myocardial infarction (MI) was developed by a joint task force among major cardiological societies, including the European Society of Cardiology (ESC), American College of Cardiology, American Heart Association, and World Heart Federation. The ESC uses the 4UD of MI in their 2018 guidelines.^5^ Peri-operative myocardial infarction associated with CABG (Type 5 MI) is diagnosed when:
  - cTn value >10 times the 99th percentile URL during the first 48 h following CABG, occurring from a normal baseline cTn value (or CK–MB >10 times the 99th percentile URL if cTn is unavailable). In addition, one of the following elements is required:
    - Development of new pathological Q-waves
    - Angiographic documented new graft occlusion or new native coronary artery occlusion
    - Imaging evidence of new loss of viable myocardium or new regional wall motion abnormality in a pattern consistent with an ischaemic aetiology
  - For cardiac surgeries other than CABG, cTn values should be considered in the context of the procedure and the extent of the expected procedural-related myocardial injury.

Other used PMI definitions come from the Society for Cardiovascular Angiography and Interventions (SCAI):

- CK–MB ≥10× URL
- CK–MB ≥5× URL and new Q-waves or left bundle branch abnormality (LBBB)
- cTn ≥70 URL
- cTn ≥35 URL and new Q-waves or LBBB
  and the Academic Research Consortium (ARC)-2:
- cTn >35× URL and new Q-waves, angiographic findings, or new regional wall motion abnormalities

Most literature on PMI focuses on patients undergoing CABG. A recent study shows that, following CABG, the 4UD and ARC-2 criteria (cardiac enzyme release plus an additional sign) remained strong predictors of all-cause mortality at 30 days and 5 years.^5^ Isolated cardiac enzyme release definitions (SCAI) were not associated with PMI relevant to prognosis. In another recent study, only high peri-operative cTn levels (well above the limit of the PMI definitions) was associated with lower long-term mortality and morbidity.^6,7^

The question remains whether the PMI definition according to the 2018 ESC guidelines (4UD) is the best we have or if we can do better. For example, it is interesting to see that all PMI definitions use cut-off values of cardiac enzymes whereas the course of the cardiac enzymes over time between ‘cardiac injury due to the procedure’ most often is different as compared with the course of the cardiac enzymes in PMI. Let us ask the experts.

## Figures and Tables

**Table 1:** Definitions of peri-operative myocardial infarction after coronary artery bypass surgery in current definitions

Definition	Year	Time after index procedure	Peak biomarker criteria	Required supporting evidence
Fourth UDMI^1^	2018	Within 48 h	cTn >10× 99th percentile URL (or CK–MB >10× 99th percentile URL if cTn unavailable)	One or more of the following: ECG: development of new pathological Q-waves; OR angiographic: new graft occlusion or new native coronary artery occlusion; OR imaging: new loss of viable myocardium or new regional wall motion abnormality in a pattern consistent with an ischaemic aetiology
ARC-2^2^	2018	Within 48 h	cTn ≥35× 99th percentile URL^a^	One or more of the following: ECG: new significant Q-waves or equivalent; OR angiographic: flow-limiting angiographic complications; OR imaging: new substantial loss of myocardium on imaging
SCAI^3^	2013	Within 48 h	CK–MB ≥5× 99th URL (or cTn ≥35× 99th percentile URL)	New pathologic Q-waves in ≥2 contiguous leads or new persistent LBBB
			CK–MB ≥10× 99th percentile URL (or cTn ≥70× 99th percentile URL)	None

ARC, Academic Research Consortium; CK–MB, creatine kinase–myocardial band; cTn, cardiac troponin; ECG, electrocardiogram; LBBB, left bundle branch abnormality; SCAI, Society for Cardiovascular Angiography and Interventions; UDMI, universal definition of myocardial infarction; URL, upper reference limit.

^a^cTn ≥70× 99th percentile with no supporting evidence is termed ‘significant peri-operative myocardial injury’ rather than myocardial infarction.

**Figure 1:**
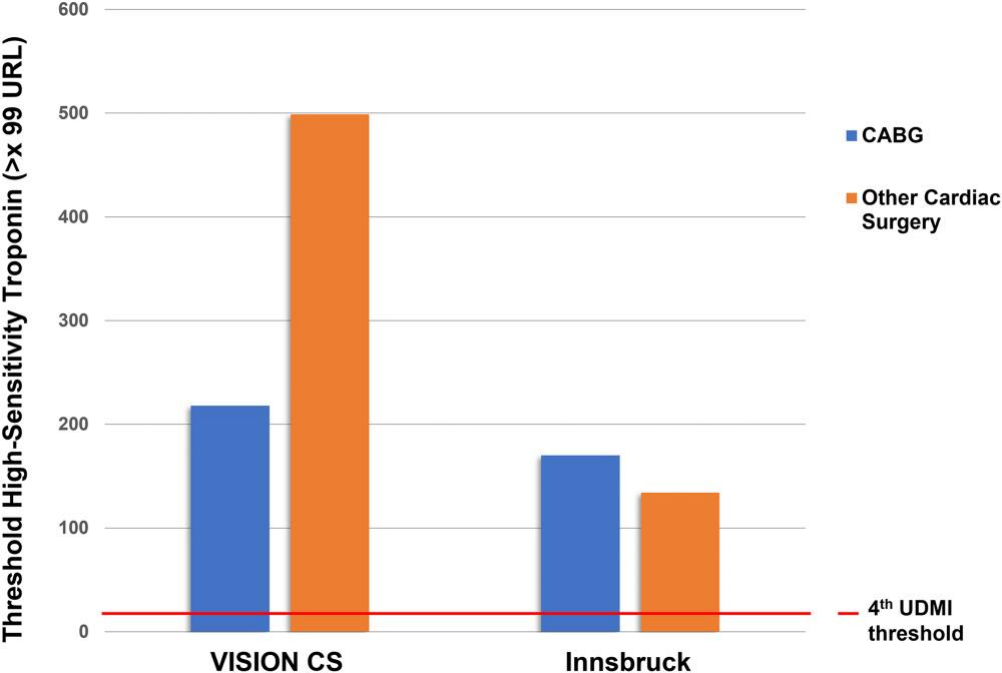
Peak cardiac troponin thresholds associated with 30-day mortality. The VISION CS Trial^14^ does not distinguish between coronary artery bypass grafting and aortic valve replacement or repair, which were included in the same coronary artery bypass grafting group with a ratio of a nearly 4:1. CABG, coronary artery bypass grafting; UDMI, universal definition of myocardial infarction; URL, upper reference limit; VISION CS, Vascular Events in Surgery Patients Cohort Evaluation Cardiac Surgery Study. See references Devereaux^14^ (VISION CS) and Pölzli^15^ (Innsbruck)

**Figure 2:**
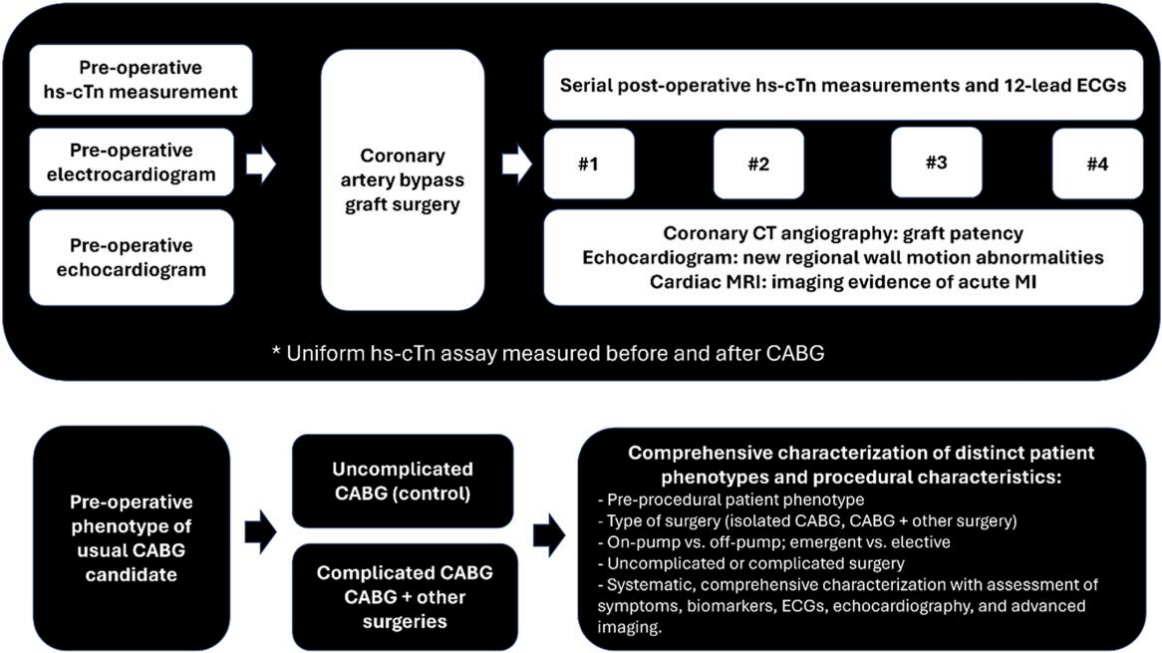
Pathway forward for defining the amount of myocardial injury induced by surgical procedures and defining how much is related to ischaemia. CABG, coronary artery bypass grafting; CT, computed tomography; hs-cTn, high-sensitivity cardiac troponin; ECG, electrocardiogram; MRI, magnetic resonance imaging

## Pro

GaudinoMario
Department of Cardiothoracic Surgery, Weill Cornell
Medicine, New York, NY, USADevereauxPhilip J
Department of Medicine, McMaster University,
Hamilton, ON, Canada
Department of Health Research Methods, Evidence, and Impact, McMaster
University, Hamilton, ON, Canada
Population Health Research Institute,
Perioperative & Surgery, Hamilton, ON, Canadahttps://orcid.org/0000-0003-0984-9011MilojevicMilan
Department of Cardiac Surgery and Cardiovascular Research, Dedinje
Cardiovascular Institute, Belgrade, Serbia
Department of Cardiothoracic Surgery, Erasmus MC,
Rotterdam, The
Netherlandshttps://orcid.org/0000-0002-1768-8433MyersPatrick O
Department of Cardiac Surgery, CHUV-Center Hospitalier Universitaire
Vaudois, Lausanne, Switzerland

‘Do the best you can until you know better. Then, when you know better, do better.’—Maya Angelou

Current definitions of peri-operative myocardial infarction (PMI) after cardiac surgery all focus on coronary artery bypass graft (CABG) surgery, with no criteria provided for PMI after non-CABG cardiac surgery procedures. With the only exception being the Society for Cardiovascular Angiography and Interventions (SCAI), all the existing PMI definitions require two key criteria for PMI diagnosis: post-operative cardiac biomarker elevation and post-operative new electrocardiographic changes. We summarize the available evidence and demonstrate how biomarker levels and the role of electrocardiographic findings in current PMI definitions should be updated. For biomarkers, we focus on cardiac troponin (cTn) as more reflective of current clinical practice and supported by more recent and higher quality evidence.

### Biomarkers

The cardiac biomarkers considered in the current PMI definitions are creatine kinase–myocardial band (CK–MB) and cTn (*Table 1*).^1–3^ Creatine kinase–myocardial band has been used for decades but has now largely been replaced by cTn in most health systems.^4^ The switch in biomarkers has generated several challenges as most of the evidence on PMI after cardiac surgery was based on CK–MB, and there are large variations in post-operative levels and dynamics between the different cTn types and between different assays for the same cTn type.^5–7^

The evidence on the association between post-operative cardiac biomarker levels and clinical outcomes has generally been consistent in showing an increase in the risk of post-operative adverse events with the increase in post-operative biomarker levels^8–12^; however, the shape and strength of this association are poorly defined, making the definition of clinically relevant thresholds highly problematic. While the finding that any post-operative increase in cardiac biomarkers has some prognostic relevance is important, from a clinical and practical perspective, it is critical to define a biomarker cut-off that separates episodes with minor vs. major impact on post-operative patients’ outcomes and, most importantly, on mortality. Unfortunately (as acknowledged by the authors), the biomarker thresholds used in current PMI definitions were chosen arbitrarily, as no rigorous evidence was available when these definitions were proposed.^1,3^

The universal definition requires an increase in peak post-operative cTn of >10× the upper reference limit (URL) for the diagnosis of PMI,^1^ while the Academic Research Consortium (ARC)-2 and SCAI definitions require an increase of ≥35× URL (≥70× URL in the SCAI definition in absence of supportive signs of myocardial ischaemia).^2,3^

However, in a *post hoc* analysis of the CABG Off- or On-Pump Revascularization Study (CORONARY) trial that included 4752 patients with CK–MB data and 1528 patients with non–high-sensitivity cTn data, at 24 and 48 h after surgery, the 10× URL threshold for cTn was reached by 46% of patients and was not associated with 30-day mortality (odds ratio 4.0, 95% confidence interval 0.8–19.3).^13^ The lower threshold of cTn associated with mortality was 130× URL.

More recently, the Vascular Events in Surgery Patients Cohort Evaluation (VISION) Cardiac Surgery study evaluated the Abbott high-sensitivity cTn assay in 13 862 cardiac surgery patients. In this large international study, the >10× URL threshold was exceeded in 97% of cases on the first post-operative day^14^; 89% of the patients exceeded the ≥35× URL threshold and 75% the ≥70× URL threshold. The 24 h cTn threshold associated with an increased risk of 30-day mortality was >210× URL after isolated CABG or aortic valve replacement and was essentially 500× URL for other cardiac surgeries. Similarly, in a large single-centre cohort study of over 8200 patients from Austria, the high-sensitivity cTn cut-offs associated with 30-day mortality varied from 41× URL for aortic valve replacement to 170× URL for CABG (*Figure 1*).^15^

Very limited data exist on the association between cardiac biomarker levels and post-operative quality of life, but the limited available data suggest that an association between PMI and quality of life outcomes is seen only for definitions that use high biomarker thresholds.^16^

In summary, recent data consistently show that the cTn thresholds used in current PMI definitions are met or exceeded in most cardiac surgery patients and that an association with adverse post-operative outcomes is only seen at cTn levels which are several times higher than those used in current PMI definitions.

### Electrocardiographic evidence of myocardial ischaemia

All the current definitions require additional signs of ischaemia (generally on the post-operative electrocardiogram) in addition to biomarker elevation for PMI diagnosis. The only exception is the SCAI definition which does not require additional ischaemic signs in case of cTn elevation ≥70× URL.

The evidence on the relationship between post-operative ischaemic electrocardiographic changes and clinical outcomes is limited and mixed, with several studies reporting a lack of association.^17^ In a study of 800 patients, post-operative biomarker elevation was significantly associated with left ventricular dysfunction and mortality independently of the electrocardiographic findings.^9^

In a review of 30 cardiac surgery studies, new conduction disturbances were reported in 3.4%–55.8% of the patients and were not associated with post-operative survival (relative risk 1.35, 95% confidence interval 0.85–2.01).^18^

In a sub-analysis of the Coronary Artery Surgery Study, new Q-waves were associated with post-operative mortality after coronary surgery^19^ and an analysis based on data from the Bypass Angioplasty Revascularization Investigation trial described similar results.^20^ However, other series reported no association between isolated post-operative Q-waves and mortality.^9,21,22^ ST-segment elevation can be detected in 10%–15% of patients after CABG, and the limited available evidence does not support an association with post-operative outcomes.^23,24^

In summary, there is very limited evidence to support an association between post-operative electrocardiographic changes and outcome in cardiac surgery patients. The inclusion of electrocardiography findings as a key part of all current PMI definitions seems based on extrapolation from what is known in patients with acute coronary syndromes or coronary artery disease and biologic plausibility, rather than data derived from cardiac surgery patients.

Although no prospective validation of those higher cTn levels has yet been published, current evidence supporting their incorporation in current PMI definition appears reasonable based on current knowledge.

### Conclusions

Recent data clearly show that the cTn cut-off adopted by all current PMI definitions is met in most cardiac surgery patients. In addition, it is clear that new post-operative electrocardiographic changes are seen in a large proportion of cardiac surgery patients and their association with clinical outcomes is weak. The low cut-off value for biomarker elevation and the reliance on electrocardiographic criteria used by current PMI definitions lead to a large overestimation of the incidence of PMI; this generates confusion in the interpretation of studies that include PMI in their outcomes and may harm patients by exposing them to unnecessary diagnostic procedures. The prognostic significance of PMI as currently defined is unclear, and this leads to uncertainty in post-operative prognostic stratification. Similar considerations have recently been published by an expert group of the European Association for Cardio-Thoracic Surgery.^25^

Thanks to recent studies and important new data, we are now in the position to define the biomarker threshold for PMI diagnosis based on solid evidence rather than on arbitrium—this is what we should do as a community. The current evidence rejects currently used criteria for PMI diagnosis and supports instead the use of a cTn threshold of at least 130–170× URL in the first 24 h, even in the absence of electrocardiographic evidence of ischaemia, as the most prognostically relevant criterion for the diagnosis of PMI after cardiac surgery.

## Contra

JaffeAllan S
Departments of Cardiology and Laboratory Medicine and Pathology and Wayne
and Kathryn Presiel Professor of Cardiovascular Disease Research, Mayo
Clinic, Rochester, MN, USASandovalYader
Minneapolis Heart Institute, Abbott Northwestern Hospital, Minneapolis
Heart Institute Foundation, Minneapolis, MN,
USAThygesenKristian
Department of Cardiological Medicine, Aarhus University
Hospital, Aarhus, Denmark

### Introduction

Myocardial injury is intrinsic to most cardiac surgical procedures including coronary artery bypass grafting (CABG). Recent data in regard to the prognosis associated with increases in cardiac troponin (cTn) after CABG have led to suggestions to change the definition of myocardial infarction after cardiac surgery proposed by the universal definition of myocardial infarction (UDMI).^1^ These sorts of calls occur when new data emerge. But however interesting and potentially important such data might seem, care should be taken in prematurely redefining clinical definitions on their basis, including peri-operative myocardial infarction (PMI).

### The original fundamentals for the redefinition of myocardial infarction

It is important to recall the original precepts that underlie the definition of myocardial infarction proposed after CABG. In 2000, when the UDMI was first proposed, several essential conceptual issues were articulated which are still important today.^2–4^ The primary precept was that a definition describes the components of a given diagnosis. It is not a treatment guideline, nor is it based on prognosis.^5^ The reasons for this will be articulated below. In the 2000 initial definition, the task force did not suggest how to make the diagnosis of myocardial infarction after percutaneous coronary intervention (PCI). With CABG, since it was known that there were unavoidable increases in cTn concentrations associated with cross-clamp time, cardiopulmonary bypass, venting of the ventricles, volume expansion, and complications from the procedure, many of which would be best characterized as due to non-ischaemic myocardial injury,^2^ the criteria suggested that PMI should only be diagnosed once one had crossed a certain cTn threshold concentration that accounted for the component of myocardial injury not due to ischaemia.^2–4^ Once that occurred, it was assumed that the additional injury was excessive and likely due to ischaemia. Cardiac troponin was selected as the favoured biomarker because it is a more sensitive and specific marker than others.^2^ The decision in regard to what that threshold of cTn should be used was arbitrary because there was a paucity of data to define what the normally expected increases post-CABG in all its iterations should be. It was well known even then that off-pump CABG caused less myocardial injury than on-pump CABG.^2^ Similarly, when concomitant valve replacement occurred, it was known that there was elaboration of more cTn.^5^ Thus, if one chose a very high threshold value based on CABG procedures using cardiopulmonary bypass, one would have had a high threshold that would have rarely diagnosed patients undergoing isolated valve replacement or having off-pump CABG as having myocardial infarction. Those with concomitant valve replacement all might meet the diagnostic threshold every time. Accordingly, an intermediate value was selected. This problem still exists today.

### The situation now

We still lack control information about the amounts of post-operative cTn associated with totally uncomplicated procedures including CABG and all its potential iterations in addition to all of the other cardiac procedures where this information is relevant. These concepts could be expanded further to PCI.

Today, there are several new studies defining threshold cTn values that provide prognostic insights. These studies are important but should not lead to challenges to the definition of PMI. It is clear that marked increases in cTn values using much more sensitive assays in patients undergoing CABG with cardiopulmonary bypass can define an early very high threshold which helps to define 30-day mortality.^2^ This is clearly an important advance because it will allow studies to evaluate whether the cause for such signals might be something that could be remediated. Specifically, one might wonder if patients with markedly increased cTn values might be enriched in the group that may have primary vascular events such as graft occlusion or coronary artery occlusion of a non-bypassed vessel that might be amenable to intervention. Unfortunately, instead of embracing this important issue, the data have led to calls to redefine PMI.

### The use of prognostic thresholds

Let us reflect for a moment about the issues related to using a prognostic cut-off to define myocardial infarction. In almost all clinical situations, the higher the cTn concentration, the more adverse the prognosis; it is a continuous relationship.^2,6,7^ There are not many patients in the cardiac surgical arena whose post-operative troponin values stay low or even normal, so we do not know how low this relationship goes in those with cardiac surgery, but in studies where the data are reported, there is a continuous relationship between prognosis and cTn concentrations.^2,6^ However, when one does cardiac surgery where one is doing a procedure with the hope that it will improve prognosis, the amount of myocardial injury (increases of cTn values) that would be adverse and prognostic needs to not only exceed baseline concentrations but be sufficient to negate the benefits of the procedure itself. Is finding a concentration threshold so high that it provides a mortality signal really the proper criteria to use to make a definition? It neglects the fact that below that threshold, there are still harmful degrees of myocardial injury because it is clear that the relationship between mortality and cTn concentrations is a continuous one even in this situation.^2,6^ It also has been reported in other studies that these lower concentrations are prognostic but over a different time course.^6^ Which timing is the best to use for a definition? Both observations are probably important, but neither should be used as a definition. In addition to issues of timing, a small amount of myocardial injury may well be critically prognostic in patients with poor ventricular function but well tolerated in those with normal ventricular function. Thus, the idea of prognosis requires not only a degree of myocardial injury but also involvement of the patient’s baseline clinical status, so it cannot be adequately applied to all individuals undergoing the procedure, all of whom have a different level of risk. One important risk factor is whether there are pre-procedural cTn increases above the 99th percentile that may identify patients with chronic myocardial injury. These increases reflect underlying heart disease. In most studies of coronary artery disease (CAD), increases in cTn identifies patients with more extensive and complex CAD.^8,9^ These patients tend to elaborate more post-operative cTn as one would expect from the more complex underlying disease.^2^ Should the same prognostic thresholds still apply? In addition, each cTn assay has a different dynamic range so that each assay is very likely to give one a very different set of values.^10,11^ This is an important consideration, but it means that taking the data from one assay to be a gold standard in the absence of data from the rest is probably not as informative as would be ideal. Finally, there are issues related to concomitant therapy which may be important. There are differences between individuals who get one sort of cardioplegia vs. another.^2^ If one is looking for a long-term outcome, there will be patients who receive guideline-directed therapy and individuals who do not and the titration becomes extremely complex. This is before one even adds the complexity of the different assays that might be involved. Finally, how one finds prognostic significance depends in part on the sample size and the endpoints. It is likely that any increases in cTn values are prognostically important, but it might take extremely large studies to ascertain that. In addition, mortality may not be the only thing that is important after cardiac surgery; graft occlusion and reduced cardiac function may all markedly impair patient well-being. Thus, all in all, it is likely impossible to identify a concentration threshold based on prognosis to define PMI. If one uses a specific threshold and it is not met despite a clinical scenario and electrocardiographic changes indicative of an acute event, should it be ignored? Even if not a mortal event, it may induce heart failure and be a critically important patient-related event.

Just think about the implications of applying this sort of thinking to the diagnosis of spontaneous myocardial infarction. No one would embrace an approach that insists on finding a downstream mortality signal post-treatment to define myocardial infarction. That said, it should be acknowledged that these prognostic signals should not be ignored. They provide an opportunity for us to delve into the possibility that there is something potentially remediable, particularly early on when we see such marked cTn increases, and they may provide information to improve the amount of myocardial injury inherent to the procedure. Clinical trialists can use these prognostic signals in their trials depending on whether they are interested in short- or long-term outcomes or both. Prognostic thresholds, once defined, could be selected based on the trial design and how much influence investigators wish these events to impart on the trial itself. Ideally, pre- and post-procedural samples would be measured in a core laboratory so that a uniform cTn assay can be deployed to reduce the difficulties of multiple different assays that often limit the comparability of cTn studies.

### The suggested solution

What is needed to move forward (*Figure 2*)? The original UDMI definition asserted that myocardial infarction requires myocardial necrosis due to ischaemia.^3,4^ In this setting, increases in cTn are a reasonable surrogate. It was also understood that there is an obligatory amount of myocardial injury that occurs during cardiac surgery.^2–4^ The mechanisms for this have recently been reviewed, and there are many.^12^ They are not all simply related to ischaemia. If one wants to define PMI after cardiac surgery, one will need first to define the amount of myocardial injury for uncomplicated procedures of each type. One might need separate criteria for on-pump CABG, off-pump CABG, isolated valve surgery, CABG plus valve surgery, and then all of the subsets, including perhaps whether myocardial injury (an increased cTn above the 99th percentile) is present pre-operatively. One could then determine what level of non-ischaemic myocardial injury is associated with uncomplicated procedures. Some believe one can use the cTn pattern to do that^13^ although that is far from proven. This is a daunting task, but the data about what one can expect from normal or abnormal procedures of a large variety of sorts could be generated. This would then allow determinations of what was above the expected and those individuals have myocardial injury, and this would generate a much better definition. One could argue that in many instances, the cause of the cTn increases may not be from acute myocardial ischaemia, particularly in the absence of other clinical criteria to support an acute myocardial infarction diagnosis, and therefore, such events should not be called myocardial infarction. These issues could be elucidated by performing appropriate imaging studies. One could then study those with more than the expected amount of myocardial injury to assess how to help identify those with ischaemia and with modern day imaging that is no longer impossible. This would be the way to refine the diagnosis. These thresholds will all vary. Those for on-pump CABG may go up, but for off-pump CABG, isolated aortic valve replacements, or transcatheter aortic valve replacement, it is hard to know. What is clear is one size will not fit all. Ideally, these data should be developed using post-operative serial samples to define the area under the time–concentration curve. These samples could then be run with multiple different assays given the marked differences in the analytical issues, dynamic ranges, and kinetics of release.^10,11^ Eventually, it may be that the criteria for some of the subsets are sufficiency similar that the number of bins for diagnosis could be reasonably collapsed into just two or three subsets. However, the data will need to guide that. For clinical trials, core laboratories could provide consistent cTn data by using only one assay. Finally, no paradigm or definition will ever be perfect. Clinical judgement is still an important component of clinical care. It is likely that the additional of clinical suspicion of events irrespective of any of the details will continue to have an important role.

### Conclusions

If this could be done, it would support the continued use of the fundamental concepts initially proposed to reach a good PMI definition. It will take work, but it would be much better than allowing the arbitrary selection of threshold values that fit some type of prognosis that will vary over time or fit the biases about how to use the PMI definition. It is not time to throw the basic concepts out; it is time to generate the information that is necessary to develop a better PMI definition. At the same time, we should not ignore the prognostic signals or the basis for the unavoidable myocardial injury. Both are areas where improvements could help patients. A similar, well thought out but not identical approach would be helpful for post-PCI injury as well. It is time to do the work and not throw out the proper concepts for expediency.
