# Correction to: Great debate: myocardial infarction after cardiac surgery must be redefined

**DOI:** 10.1093/eurheartj/ehae734

**Published:** 2024-10-11

This is a correction to: Mario Gaudino, Allan S Jaffe, Milan Milojevic, Yader Sandoval, Philip J Devereaux, Kristian Thygesen, Patrick O Myers, Jolanda Kluin, Great debate: myocardial infarction after cardiac surgery must be redefined, *European Heart Journal*, 2024, ehae416, https://doi.org/10.1093/eurheartj/ehae416

In the originally published version of this article, there was a typographic error in author Philip J Devereaux's name. This error has been corrected.

